# Millets Can Have a Major Impact on Improving Iron Status, Hemoglobin Level, and in Reducing Iron Deficiency Anemia–A Systematic Review and Meta-Analysis

**DOI:** 10.3389/fnut.2021.725529

**Published:** 2021-10-14

**Authors:** Seetha Anitha, Joanna Kane-Potaka, Rosemary Botha, D. Ian Givens, Nur Liana Binti Sulaiman, Shweta Upadhyay, Mani Vetriventhan, Takuji W. Tsusaka, Devraj J. Parasannanavar, Thingnganing Longvah, Ananthan Rajendran, Kowsalya Subramaniam, Raj Kumar Bhandari

**Affiliations:** ^1^Smart Food Initiative, International Crops Research Institute for the Semi-Arid Tropics (ICRISAT), Patancheru, India; ^2^Development Strategy and Governance Division, International Food Policy Research Institute (IFPRI), Lilongwe, Malawi; ^3^Institute of Food, Nutrition and Health, University of Reading, Reading, United Kingdom; ^4^UNICEF, Lilongwe, Malawi; ^5^Ostrom Center for Advanced Studies on Natural Resources Governance, Asian Institute of Technology, Pathumthani, Thailand; ^6^National Institute of Nutrition (NIN), Hyderabad, India; ^7^Food Science and Nutrition, Avinashilingam Institute for Home Science and Higher Education for Women, Coimbatore, India; ^8^National Technical Board of Nutrition, Government of India (GoI), New Delhi, India

**Keywords:** iron status, hemoglobin level, millets, sorghum, meta-analysis

## Abstract

The prevalence of iron deficiency anemia is highest among low and middle-income countries. Millets, including sorghum, are a traditional staple in many of these countries and are known to be rich in iron. However, a wide variation in the iron composition of millets has been reported, which needs to be understood in consonance with its bioavailability and roles in reducing anemia. This systematic review and meta-analysis were carried out to analyze the scientific evidence on the bioavailability of iron in different types of millets, processing, and the impact of millet-based food on iron status and anemia. The results indicated that iron levels in the millets used to study iron bioavailability (both *in vivo* and *in vitro*) and efficacy varied with the type and variety from 2 mg/100 g to 8 mg/100 g. However, not all the efficacy studies indicated the iron levels in the millets. There were 30 research studies, including 22 human interventions and 8 *in vitro* studies, included in the meta-analysis which all discussed various outcomes such as hemoglobin level, serum ferritin level, and absorbed iron. The studies included finger millet, pearl millet, teff and sorghum, or a mixture of millets. The results of 19 studies conducted on anaemic individuals showed that there was a significant (*p* < 0.01) increase in hemoglobin levels by 13.2% following regular consumption (21 days to 4.5 years) of millets either as a meal or drink compared with regular diets where there was only 2.7% increase. Seven studies on adolescents showed increases in hemoglobin levels from 10.8 ± 1.4 (moderate anemia) to 12.2 ± 1.5 g/dl (normal). Two studies conducted on humans demonstrated that consumption of a pearl millet-based meal significantly increased the bioavailable iron (*p* < 0.01), with the percentage of bioavailability being 7.5 ± 1.6, and provided bioavailable iron of 1 ± 0.4 mg. Four studies conducted on humans showed significant increases in ferritin level (*p* < 0.05) up to 54.7%. Eight *in-vitro* studies showed that traditional processing methods such as fermentation and germination can improve bioavailable iron significantly (*p* < 0.01) by 3.4 and 2.2 times and contributed to 143 and 95% of the physiological requirement of women, respectively. Overall, this study showed that millets can reduce iron deficiency anemia.

## Introduction

Iron deficiency anemia is a serious global public health problem. As per the World Health Organization (WHO) report, worldwide, 42% of pregnant women, 30% of non-pregnant women (aged 15–50 years), 47% of preschool children (< 5 years), and 12.7% of young men (> 15 years) are anaemic. Iron deficiency anemia (IDA) adversely affects the growth and cognitive development in children; cognitive, physical, and psychological health in non-pregnant women, and maternal and neonatal outcomes in pregnant women. Its prevalence among women between the ages of 15 and 49 is more than 40% in most Asian and African countries (1). Many factors cause IDA including, gut health, dietary iron deficiency, bioavailability, folic acid deficiency, Vitamin C, Vitamin A, and Vitamin B12 deficiency. In addition, hookworm infestation and malaria also contribute to the increase in the prevalence of IDA among Asian and African countries (2).

Three major approaches are followed to control IDA globally, which are supplementation with iron and folic acid tablets, fortification and natural food-based approaches. Despite the wide implementation of the first two approaches, IDA remains a serious malnutrition problem with an increasing trend globally. The third approach mainly focuses on dietary diversification and enrichment of diets with naturally iron-rich foods without the potential side effects of artificial additives.

In developing countries, milled rice, wheat, and maize replaced the traditional nutritious crops. Refined foods are abundant in starch but lack nutrients, especially micronutrients such as iron (Fe) and zinc (Zn). Given that a major part (>80%) of the diet in developing countries (3) comprises low iron staple food, achieving sufficient intake of iron through the remaining 20% of the diet is impractical. Therefore, it is important to diversify the staple food by including naturally iron-rich food crops such as millets (4). In addition, millets have a 2.3 to 4.0 times more dietary fiber (6.4 ± 0.6 to 11. 5 ± 0.6 g/100g) compared with refined rice and refined wheat (5), which acts as food for beneficial gut microbiome that improves abundance and alters the gut composition in a beneficial way (6, 7). Millets have added advantages as they are recognized as a smart food, i.e., not only good for you since it is nutritious and healthy, but also good for the planet because it is environmentally sustainable and good for farmers since it is resilient and climate-smart (8).

Animal sources of haem iron are well known for their high bioavailability. However, it is not always affordable to the poorest segments of the population. Moreover, a vegetarian population require alternate plant sources of highly absorbable iron to tackle iron deficiency. Although millets are recognized as being naturally rich in iron, their nutrient composition varies with the type, variety, and growing conditions. Commonly used food composition tables while providing an overview of the nutrient composition do not include this detail (5, 9, 10). Presenting a single value for iron level in a type of millet can be misleading as iron levels can vary significantly among varieties. Iron levels can be as much as triple in a commonly available variety over another.

Non-haem iron (plant-based) is not absorbed as readily as haem iron (animal-based) in the presence of phytate and tannin in millets. Most of the cereals such as wheat flour, brown rice, and barley contain phytic acid to levels (5) that are far higher than that of millets. However, the phytate content of millets is often overly emphasized. Nonetheless, it is important to understand the bioavailability of iron from millets and its impact on anemia status. Although few studies have investigated the bioavailability of iron by *in vitro* and *in vivo* methods, not all of them are well known or promoted. Moreover, very few studies focus on the overall beneficial effects of millets on anemia, as most studies focus on only one or just a few of the outcomes, such as hemoglobin, absorbed iron, serum ferritin, and serum transferrin levels. Collating this information can provide information on which millets to use and to what extent they can improve iron status and the type of processing that can enhance the bioavailability of dietary iron. It is against this background that this systematic review of published scientific studies on millets was undertaken to investigate the range of iron levels and its bioavailability in order to enable a comparison with major staples such as rice, wheat (both whole grain and refined), and maize. This was followed by a meta-analysis to collate all the science-based evidence available on millets, their effects on iron status, hemoglobin levels in the body, and their related ability to reduce iron deficiency anemia.

### Research Questions

Does consumption of millets-based food help improve iron status and hemoglobin levels and reduce iron deficiency anemia? How does this compare with regular non-millet diets?

## Methods

### Study Period and Protocol

The systematic review and meta-analysis were conducted from October 2017 to April 2021. The PRISMA checklist was used to write the protocol (11). The protocol was registered through an online “research registry” with the Unique Identification number “reviewregistry1114”.

### Search, Inclusion, and Exclusion Criteria

Studies written in English and published between 2010 and 2020 were considered. Google Scholar, Scopus, Web of Science, PubMed, and CABI abstract were used to find studies relevant to the research questions. The search was conducted using the search strategy and keywords (Table 1), which were further screened for relevance to the study, completeness of information and quality of research based on the inclusion and exclusion criteria.

**Table 1:** Search keywords used to identify relevant papers.

**Number**	**Search keywords**
1	Millets efficacy in reducing anemia
2	Millets “AND” bioavailability of iron
3	Impact of consuming millets on iron status/hemoglobin level
4	Efficacy of millets on hemoglobin level. Repeat the search by replacing the word “millets” with “finger millet,” “pearl millet,” “sorghum,” teff.
5	Efficacy of millets on iron deficiency anemia
6	Effect of consuming millets on ferritin level
7	Effect of germination of millets on iron bioavailability. Repeat the search by replacing the word “millets” with “finger millet,” “pearl millet,” “sorghum,” teff. Repeat the search by replacing the word “germination” with “fermentation,” “malting,” “processing.”
8	Hand search on the references of published article.

### Inclusion Criteria

The following criteria were included: (1) research studies conducted to test the efficacy of millets in reducing anemia and improving hemoglobin, serum ferritin levels, iron status, and/or bioavailability of iron; (2) studies that had information on any or all of the outcomes including levels of hemoglobin, serum ferritin, absorbed iron, and bioavailability of iron; (3) efficacy studies conducted using high iron and/or biofortified varieties of millets; (4) studies conducted on any age group or gender of any population to test the efficacy of millets in reducing iron deficiency; (5) both *in vivo* and *in vitro* studies that assessed the bioavailability of iron, with the two types of studies treated as separate; (6) peer-reviewed journal articles, full MSc or PhD theses submitted to universities, and full research papers from theses if available online.

### Exclusion Criteria

Review articles, animal studies, and publications with incomplete data were excluded.

### Data Collection

**Figure 1:**
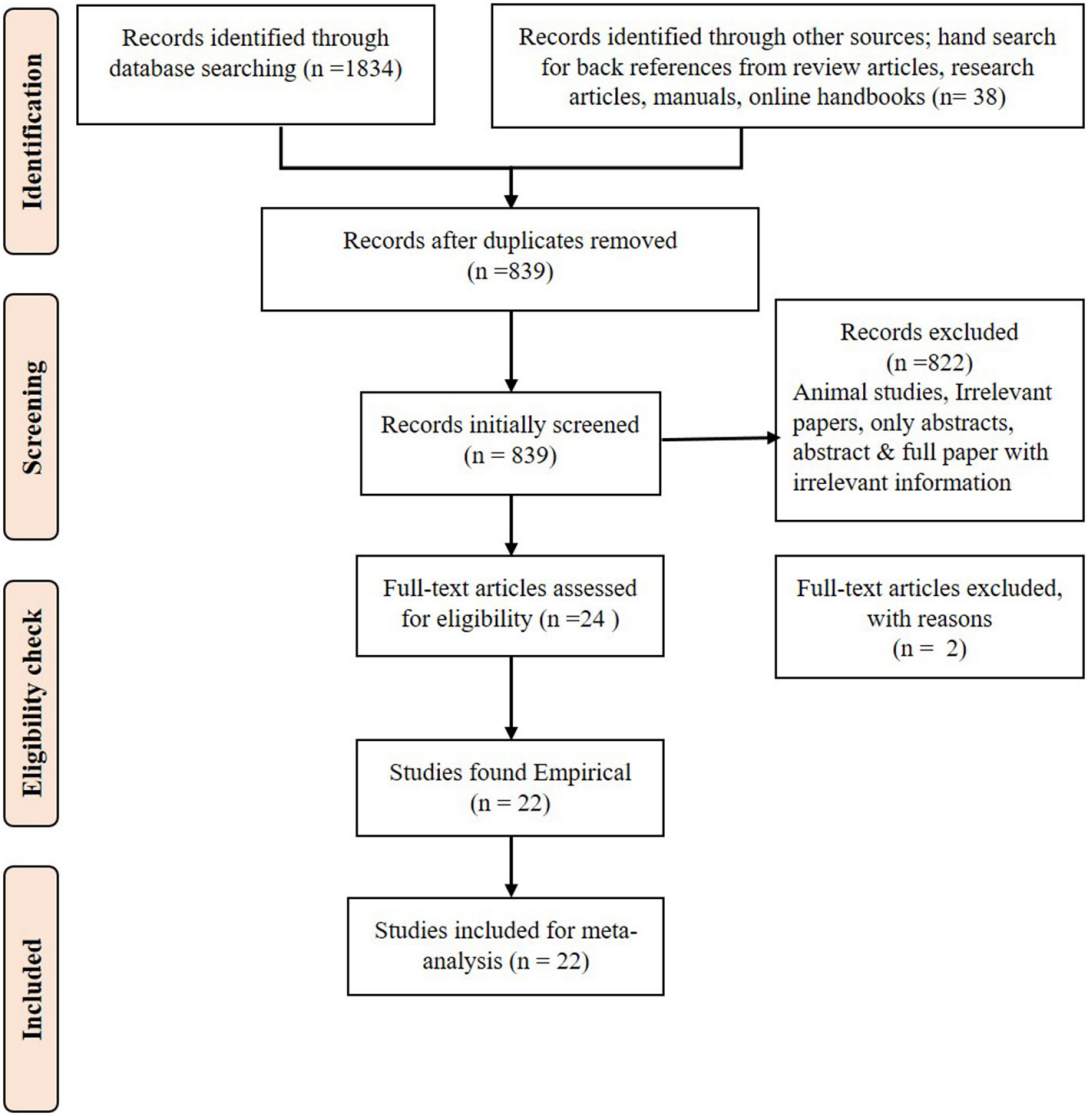
PRISMA flow diagram for systematic review.

The study used the PRISMA checklist at every step of data collection, extraction, and analysis (Figure 1). Only the relevant papers downloaded that addressed the research questions were used. The references in the relevant publications were also checked by hand search to find more related research articles. If only an abstract was found relevant to this study, then efforts were made to download open access articles or collect the full paper. After collecting the full paper, if any required data were missing, the authors were contacted, and the full information was requested for use in the meta-analysis. The data were extracted from the articles and documented in Excel sheets. Using the data, descriptive statistics, regression analysis, forest plots, and publication bias analysis were performed.

### Data Items

Each study was labeled with details on the author and the year of publication. The age group and gender of the study participants were recorded, along with the country, study method, sample size, and type and form of millets used. The numerical variables considered for the meta-analysis included mean and standard deviations of levels of hemoglobin, absorbed iron, and serum ferritin. The data were then entered into an Excel sheet as per the guidelines (12, 13).

### Summary Measures and Result Synthesis

(1). Pre-and post- intervention or (2). test and control diets impact on each outcome was recorded with mean and standard deviation values and used for meta-analysis. Since it is continuous data, a meta-analysis was performed to measure Standardized Mean Difference (SMD) and heterogeneity (i^2^). The significance of the results was determined using the fixed-effect model for a single source of information and the random effect model for other studies. Results from both models were captured in each forest plot. In addition, descriptive statistics such as mean, standard deviation, and percentage change in hemoglobin levels were calculated for both intervention and control samples. Regression analysis was conducted to test the effects of processing such as germination, fermentation, and malting of millets on the bioavailability of iron. The term ‘bioavailability' was used to refer to the percentage of iron in the food that is apparently absorbable based on the *in vivo* and *in vitro* protocols used in the studies included in the meta-analysis. The term ‘bioavailable iron' was used to represent the amount of apparently absorbed iron per 100 g of food, and has been calculated as:

Bioavailable iron (mg/100 g food) = iron concentration in the food (mg/100 g) × % bioavailability/100.

Bioavailable iron from millets was then compared with the physiological requirement, which is a requirement for absorbed iron (14). The physiological requirement for various age groups to assess whether the bioavailable iron from millets can contribute to the physiological requirement. The physiological requirement of iron was obtained from the recommended dietary allowances book released recently (15) and was calculated based on the assumption that 8% of the iron is absorbed from the Estimated Actual Requirement (EAR) (15).

### Study Quality Assessment

Using the eight-item Newcastle-Ottawa Scale (NOS), the quality (16, 17) of each study was assessed by two investigators. Any disagreement was resolved by discussing it with a third reviewer. The researchers also applied the principle stated in the study of (18) to further strengthen quality assessment.

### Detailed Data Analysis

A total of 22 human studies were found eligible for the meta-analysis with three outcomes namely hemoglobin level (g/dl), serum ferritin (ng/ml) and total iron absorbed (mg/day). Nineteen of these studies (based on various types and forms of millets) were used to determine the effects of consumption of millets on hemoglobin levels, while two studies were used to determine the effects on iron absorption, and four studies were used to measure the effects on serum ferritin levels (a blood protein that contains iron that is commonly tested to indicate the level of iron stored in the body). The iron content of millets was categorized as high if iron content was above 6 mg/100 g (regardless of biofortification), moderate it was from 3 mg/100 g to 6 mg/100 g, and low if below 3 mg/100 g. They were compared with the corresponding control samples which were mostly rice or wheat-based regular diets as well as low iron millets. The heterogeneity of samples and overall test results were included in the forest plots. Both the random effect and fixed-effect models were tested and used to interpret the results and SMD (19–22).

A meta-analysis was conducted using R Studio 4.0.4 (2021) (www.rstudio.com) to obtain a forest plot, heterogeneity, overall test effects in both fixed and random effect models, and funnel plots to determine the publication bias (12, 23).

### Subgroup Analysis

Based on the type of millet (finger millet, pearl millet, sorghum, and mixed millets), the duration of the study (‘short' if <4 months while ‘long' if > 4 months) and the age of the participants (children, adolescents, and adults) were used for subgroup analysis to assess the effects of consumption of millets on hemoglobin levels.

### Risk of Bias Assessment

A funnel plot was used to assess publication bias. Selection, detection, attrition and reporting biases were assessed according to the guidelines provided in the Cochrane handbook for systematic reviews of interventions (24).

## Results

### Meta-Analysis of Data From Human Studies

There were 22 research papers involving human subjects identified as eligible for the meta-analysis for three outcomes, namely, hemoglobin levels, serum ferritin levels, and total iron absorbed.

### Hemoglobin Level

**Figure 2:**
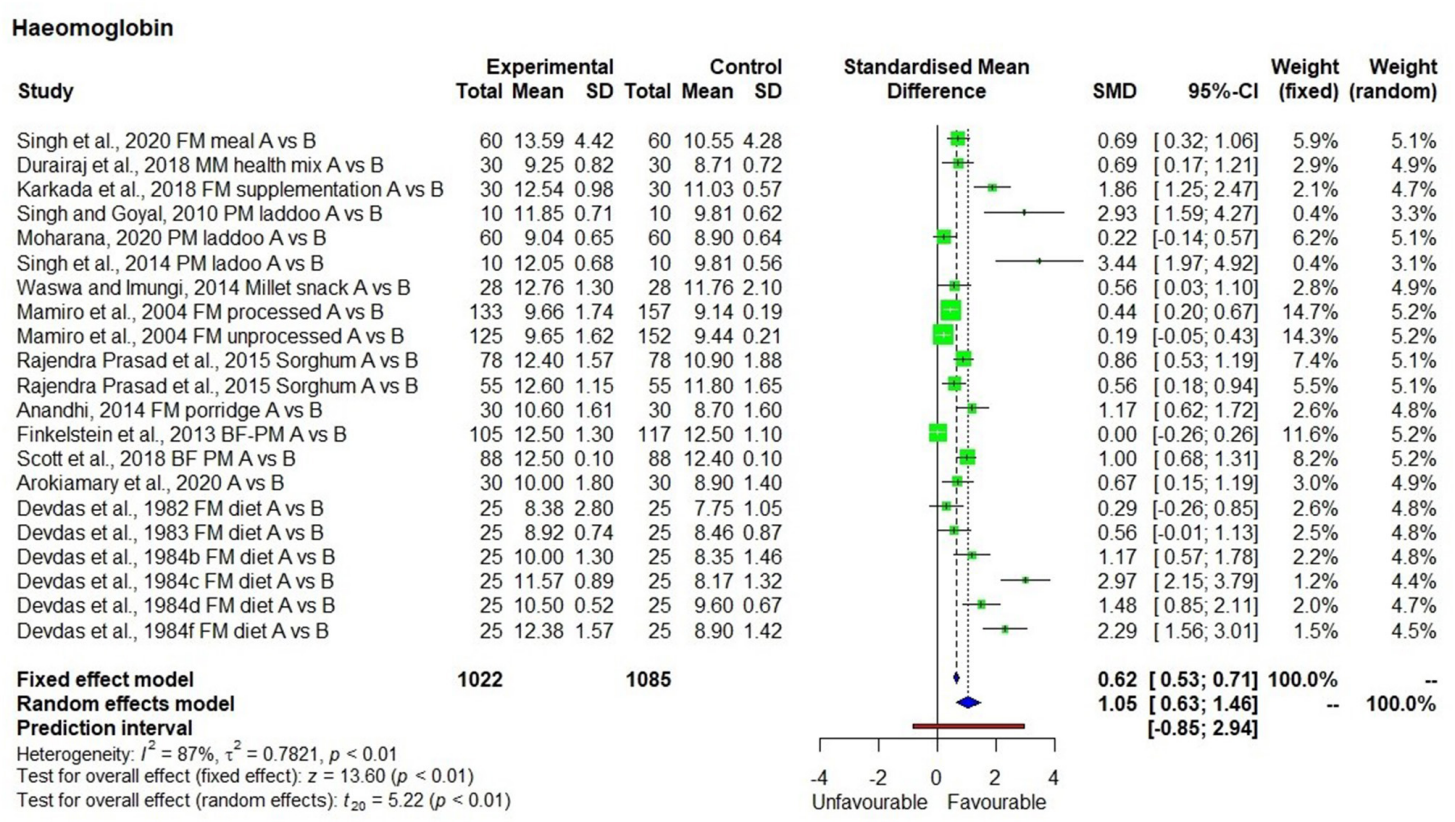
Effect of consuming millets on Hemoglobin level.

There were 19 studies (25–43) used to conduct the meta-analysis on hemoglobin levels, which showed high heterogeneity (I^2^ = 87%) and statistical significance (Figure 2). The hemoglobin levels in 1,022 individuals (from 19 studies) produced SMD of 1.05 at a 95% confidence interval (CI) ranging from 0.63 to 1.46 indicating a significant (*p* < 0.01) overall improvement in hemoglobin levels within the group that had consumed millets for a period ranging from 28 days to 4.5 years. On average, there was a 13.2% increase in hemoglobin levels relative to the baseline in the intervention group who received millet supplementation which is five times higher compared with only a 2.7% increase in hemoglobin levels in the control group who did not receive millet supplementation and were consuming regular rice or wheat-based diet. Seven studies conducted on adolescents showed an increase in hemoglobin levels from 10.8 ± 1.4 (moderate anemia) to 12.2 ± 1.5 g/dl (normal). The studies that qualified for the meta-analysis used finger millet, pearl millet, sorghum, or mixed millets (kodo, little, and foxtail millets). Among the 19 studies, 2 studies used pearl millet which had an iron content averaging 8.6 mg/100 g (44, 45), while the rest of the studies did not indicate the iron content of millets used in meal preparation.

### Iron Absorption

**Figure 3:**
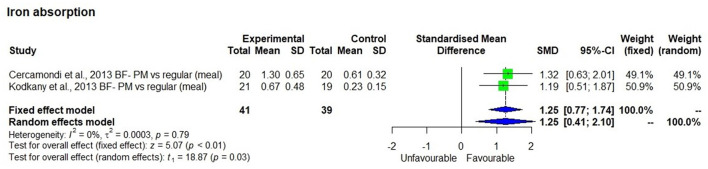
Effect of consuming millet based meal on bioavailable iron content compared to regular meal.

The meta-analysis using two studies (44, 45) that measured iron absorption showed that bioavailable iron in the study that had used high iron pearl millet (8.2 mg/100 g grain) was significantly (*p* < 0.01) higher (Figure 3) than in the one that had used low iron millets (< 3 mg/100 g grain) with SMD of 1.25 and 95% CI of 0.77 and 1.74, respectively. The bioavailable iron was 1 ± 0.45 mg/day from a dietary iron intake of 14.1 mg/day compared with 0.42 ± 0.27 mg/day from a dietary iron intake of 6.3 mg/day, which is 7.5 ± 1.6 % bioavailability.

### Serum Ferritin Level

**Figure 4:**
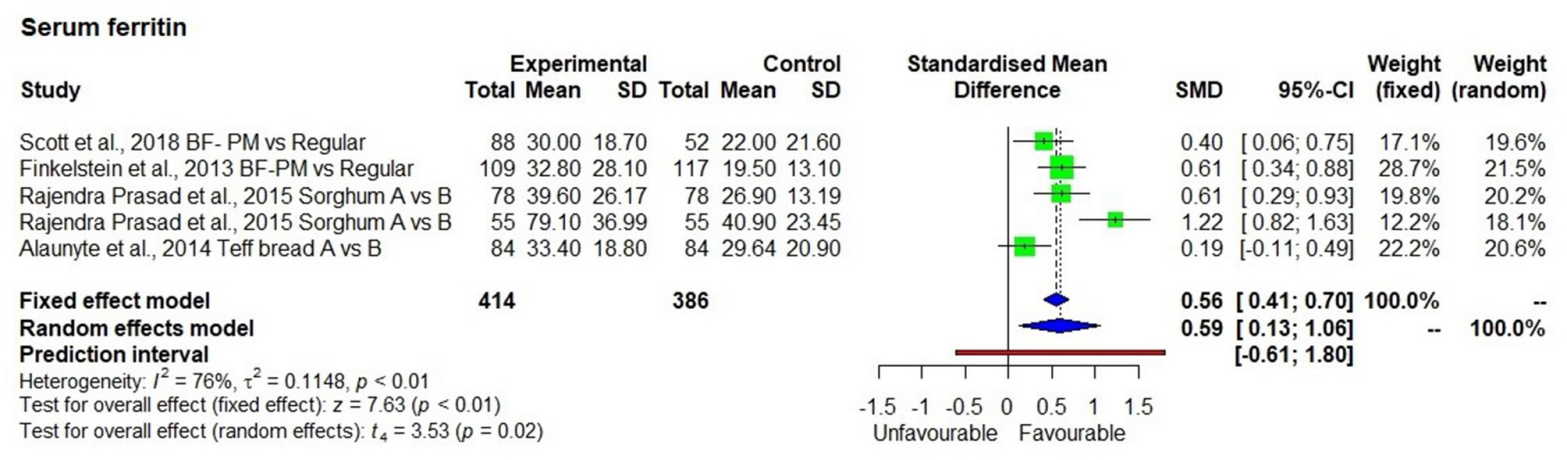
Effect of consuming millet based meal on serum ferritin level.

Four studies (33, 35, 36, 46) measured serum ferritin, which was significantly increased in groups consuming high iron millet-based meals (Figure 4), compared with low iron millet-based meals or non-millet-based meals (*p* < 0.01) with moderate heterogeneity among the studies (I^2^ = 76%) and SMD of 0.59 and 95% CI of 0.13; 1.06.

There was a 54.7% average increase in serum ferritin levels after the consumption of pearl millet (two studies), sorghum meals (one study), and teff bread (one study). The average iron level in pearl millet-based meal was 8.2 mg/100 g while it was 5.6 mg/100 g in teff bread. The intervention was conducted for 6 weeks using teff bread and 6 months using pearl millet-based meals. The iron levels in sorghum used in the study for 8 months were not indicated.

### Meta-Analysis of *in-vitro* Iron Bioavailability Studies

**Figure 5:**
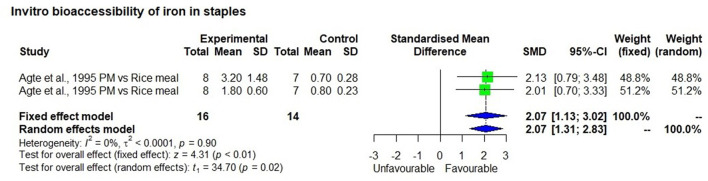
In-vitro bioavailability of iron in millet compared to rice based meal.

**Figure 6:**
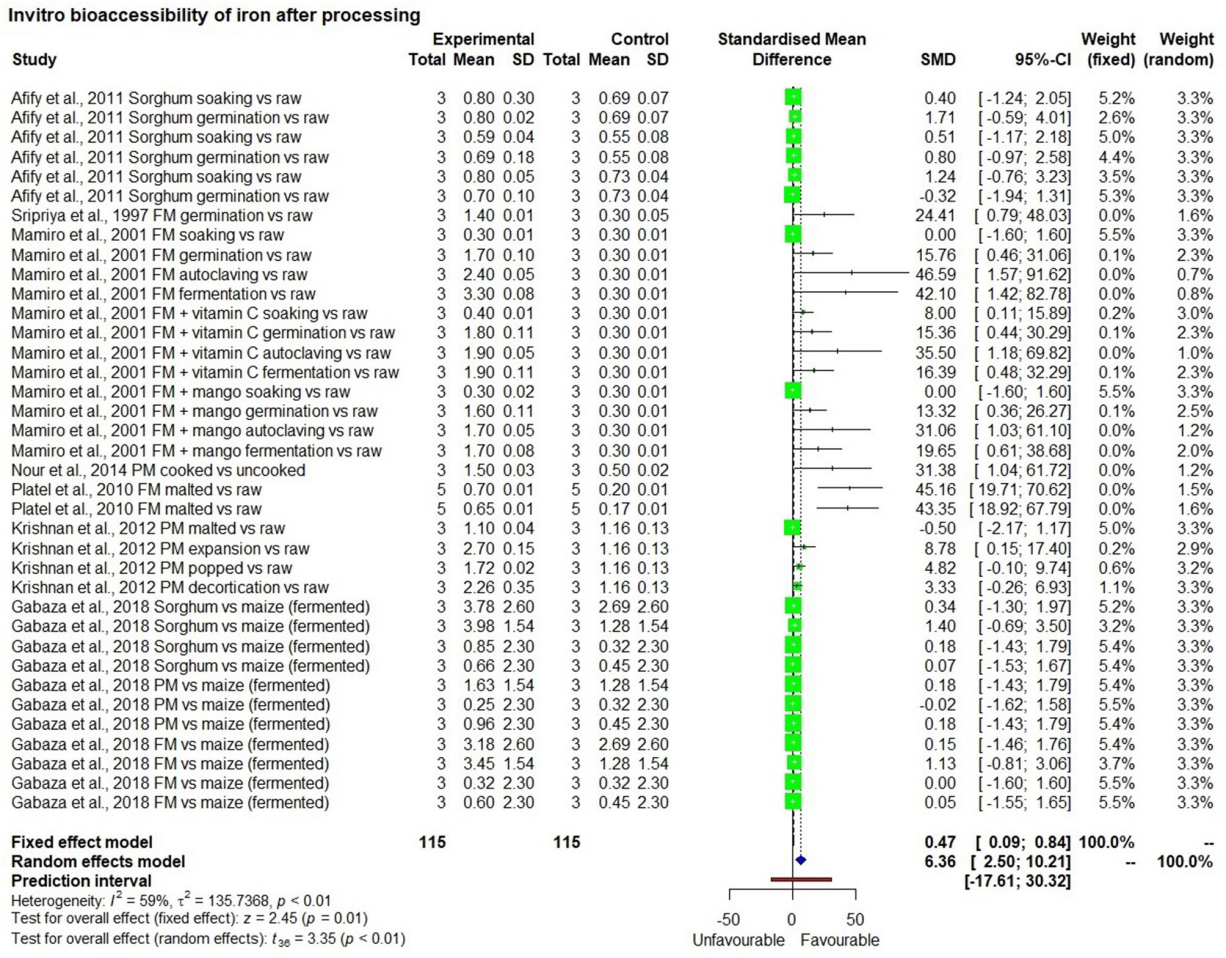
In-vitro bioavailability of iron in millets after processing.

The meta-analysis included eight studies that measured *in vitro* iron bioavailability in pearl millet and the effects of processing. One study with two observations (47) showed 2.5 mg/100 g bioavailable iron in a high iron pearl millet-based meal, which was significantly higher (*p* < 0.01) than in the rice-based control meal (0.75 mg/100 g) with a bioavailability percentage of 7.5% and 7.9%, respectively (Figure 5). Similarly, seven *in-vitro* bioavailability studies showed (48–54), that processing such as germination, fermentation, decortication, expansion (a thermal process that increases the size and volume of the grain) and popping of millet grains had significantly (*p* < 0.01) increased bioavailable iron than those in unprocessed control millet grain (Figure 6). The increase in bioavailable iron by fermentation and germination was 3.4 times and 2.2 times higher, respectively than in unprocessed millets.

### Additional Statistics

**Figure 7:**
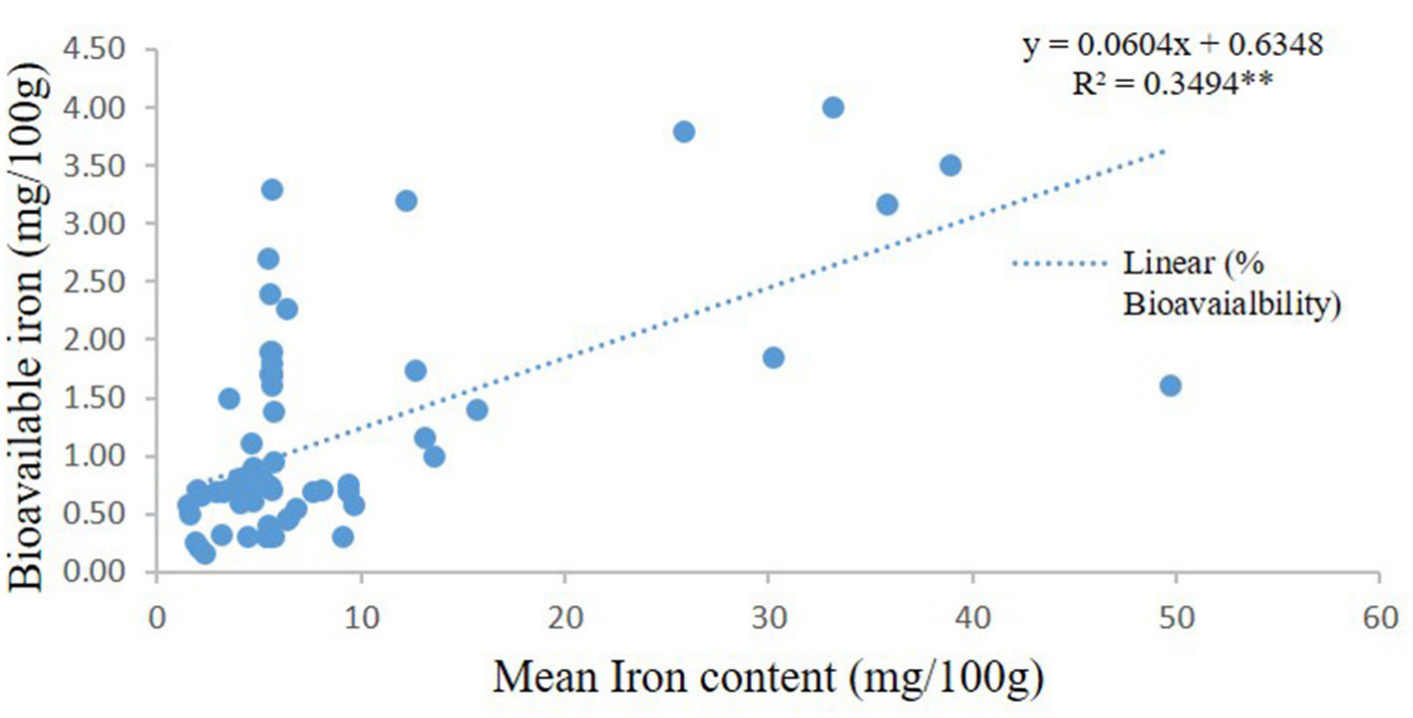
In-vitro bioavailable iron from millets.

Statistical comparison was conducted to determine the percentage bioavailability of iron and bioavailable iron in high iron pearl millet in two *in vivo* studies (44, 45) and 11 *in vitro* studies on pearl millet, finger millet, and sorghum (47–57). The iron content in the millets used in the *in vitro* studies was 17.27 ± 13.38 mg/100 g. The *in-vitro* studies also showed a significant increase in bioavailable iron (mg/100 g) with the increasing iron content of millets (Figure 7). It is also noted some studies shows high iron content in the fermented millets even up to 49.7 mg/100 g (54). On the other hand, based on the two human studies conducted using iron-rich pearl millet (8.3 mg/100 g) showed the bioavailability of 7.5 ± 1.6% with bioavailable iron of 1.0 ± 0.4 mg/100 g, while the concentration of iron in the entire pearl millet-based meals was 14.1 ± 9 mg/100 g.

Regardless of the iron concentration in millets, the overall percentage bioavailability in millets from human studies was 7.22 ± 1.78%, with an overall bioavailable iron content of 0.7 ± 0.45 mg/100 g (Table 2).

**Table 2:** Iron bioavailability and bioavailable iron in millets based on the type of study (*in vitro* and *in vivo*).

**Type of study**	**Iron content (mg/100 g; mean ± SD)**	**Iron bioavailability (%) (mean ± SD)**	**Bioavailable iron (mg/100 g; mean ± SD)**
*In vitro*	8.26 ± 9.31	17.64 ± 12.49	1.13 ± 0.95
*In vivo*	9.8 ± 6.4	7.22 ± 1.78	0.70 ± 0.40

In this systematic review, the iron content in millets, regardless of biofortification, were organized into low (<3 mg/100 g), moderate (3 to <6 mg/100 g), and high (6 mg/100 g) categories based on their provisions of >30% (high), 15–30% (moderate), and <15% (low) daily iron requirements for adults. The bioavailability from these categories was assessed. The results showed that meals prepared with high iron millets had high bioavailable iron that can provide 100% of the physiological requirement of iron as proposed by ICMR (2020) (Table 3). Results from *in vivo* studies showed that bioavailable iron was high in high iron millets (1 ± 0.4 mg/100 g), compared with low iron millets (0.4 ± 0.2 mg/100 g).

**Table 3:** The effect of processing on the bioavailability of iron in millets and its potential to meet the physiological requirement of iron.

**Process**	**Mean iron content (mg/100 g)**	**Bioavailability (%)**	**Bioavailable iron (mg/100 g)**	**Contribution to the physiological requirement of iron (%)** **(ICMR, 2020)**							
				**1–3 yrs**	**4–6 yrs**	**7–9 yrs**	**10–12 yrs girls**	**13–15 yrs girls**	**16–17 yrs girls**	**Adult men**	**Adult women**
***In vivo*** **human study**											
Boiled/baked meal	6.3 ± 3.1	6.0 ± 1.9	0.4 ± 0.3	105	82	66	31	29	27	44	33
Boiled/baked meal	12.6 ± 6.9	8.3 ± 1.1	1.0 ± 0.4	263	204	164	79	72	68	110	83
***In vitro*** **method**											
Raw/unprocessed grain	5.4 ± 2.9	9.8 ± 6.8	0.5 ± 0.3	124	96	77	37	34	32	52	39
Boiled meal	23.2 ± 17.2	19.1 ± 20.3	2.2 ± 0.9	570	442	355	171	156	148	238	181
Decorticated grain	4.2 ± 3.2	24.8 ± 15.7	1.3 ± 1.4	332	257	207	99	91	86	139	105
Dephytinized grain	3.3 ± 1.7	24.6 ± 8.7	0.7 ± 0.2	190	148	119	57	52	50	79	60
Fermented grain	14.6 ± 13.8	15.4 ± 13	1.7 ± 1.3	452	351	282	135	124	118	189	143
Germinated grain	5.2 ± 1.0	22.0 ± 8.2	1.1 ± 0.5	300	233	187	90	82	78	125	95
Malted flour	2.9 ± 1.4	28.0 ± 3.5	0.8 ± 0.2	215	167	134	64	59	56	90	68
Popped grain	12.7 ± 0.21	13.4^*^	1.73 ± 0.0	455	353	284	136	124	118	190	144
Expanded grain	5.5 ± 0.4	49.1^*^	2.7 ± 0.2	711	551	443	213	194	185	297	225
Soaked grain	4.9 ± 0.7	11.6 ± 6.1	0.5 ± 0.2	140	109	87	42	38	36	58	44

* *SD values not available. All in vitro studies are based on 100 g of grain, whereas in vivo studies, they varied from 84 g to 300 g (44, 45, 47–50, 53–55, 57, 58). The quantity required to be consumed can be adjusted to meet 100% of the physiological requirement*.

### Effects of Processing on the Bioavailability of Iron

Processes such as fermentation, germination, and soaking did not affect the total iron content of the grain significantly. Moreover, there was significantly higher bioavailable iron in these processes compared with that in unprocessed millets (Table 3). On the other hand, decortication, popping, and malting reduced the iron content. However, bioavailable iron was still higher than or equal to that in unprocessed grains.

Millets can provide 100% of the physiological requirement of iron for different age groups (45), though this depends on the type, variety, and the kind of processing undergone. Based on the results from *in vitro* studies, soaking was not significantly associated with an increase in iron bioavailability (Table 3). On the other hand, even if the iron content in millets was low, malting increased bioavailable iron by 3.5 times, and thereby bioavailable iron was 1.6 times higher than in unprocessed grains which increased its contribution to the physiological requirement to 1.7 times in adult women (from 39 to 68%). There were 17 observations on fermented millets which showed that the fermentation process did not affect the iron content of grains. However, it increased bioavailable iron by up to 3.4 times (from 0.5 to 1.7 mg/100 g) and can help increase the contribution to the physiological iron requirement by 3.4 times in adult women (from 39 to 143%) (Table 3). Fermentation was found to be superior to germination and malting. Germination increased bioavailable iron content by up to 2.2 times compared with unprocessed grains (from 0.5 to 1.1 mg/100 g) and helped meet 95% of the physiological requirement in adult women, which is 2.4 times higher than in unprocessed grains. Adding an absorption enhancing agent such as Vitamin C rich food improved the percentage of iron bioavailability by up to 6.8 times (50). Other processes such as decortication and dephytination using phytase enzyme are industrial processes. While both processes decreased iron content in grains by more than 50%, they increased bioavailable iron by 2.6 and 1.4 times, respectively, thereby increasing their contribution to the physiological requirement in adult women by 2.7 and 1.5 times, respectively.

Popping slightly reduced (3%) the iron content of grains. However, it increased bioavailable iron by 3.4 times and thereby increasing its contribution to the physiological requirement by 3.7 times. Compared with popping, expansion led to a loss of more than 60% of grain iron content while increasing bioavailable iron by 5.4 times compared with unprocessed grains and thereby increased the % contribution to the physiological requirement by 5.8 times in adult women.

It may be noted that processing did not have the same impact in all the studies, possibly due to the difference in the methods used, which needs further evaluation. Fermentation increased mean bioavailable iron content in millets more than all other processing methods.

Three studies conducted on the effects of processing on phytate content showed a reduction in phytate content by 29.7% after germination, 28.1% after soaking, 30.7% after decortication, and 51% after expansion (Table 4). This reduction in phytate content increased the bioavailability of iron in these studies. Decortication increased bioavailable iron content by 160% (0.5 mg/100 g to 1.3 mg/100 g).

**Table 4:** The effects of processing on phytate and tannin content in millets (mg/100 g).

**Type of grain**	**Raw**	**Germination**	**Soaking**	**Decortication**	**Cooking**	**Expansion**	**Popping**
**Phytate**							
Sorghum	584.5 ± 25.4	410.5 ± 15.8	420.3 ± 9.2				
Finger millet	529.0 ± 0.3			363.0 ± 0.2	-	259.0 ± 0.5	549.0 ± 0.5
Pearl millet	203.0 ± 7.25				175.28 ± 2.54		
Reduction in phytate (%)		29.7	28.1	30.7	13.6	51.0	−3.8
**Tannin**							
Pearl millet	19.0 ± 0.0				18.0 ± 0.0		
Finger millet	973.9 ± 23.0	482.6 ± 12.6					
Reduction in tannin (%)		50.4			5.2		

Germination of finger millet increased bioavailable iron content from 0.4 to 1.3 mg/100 g and decreased tannin content by 50.4%. In pearl millet, cooking reduced tannin content by 5.2% (Table 4).

### Subgroup Analysis by Type of Millet

**Figure 8:**
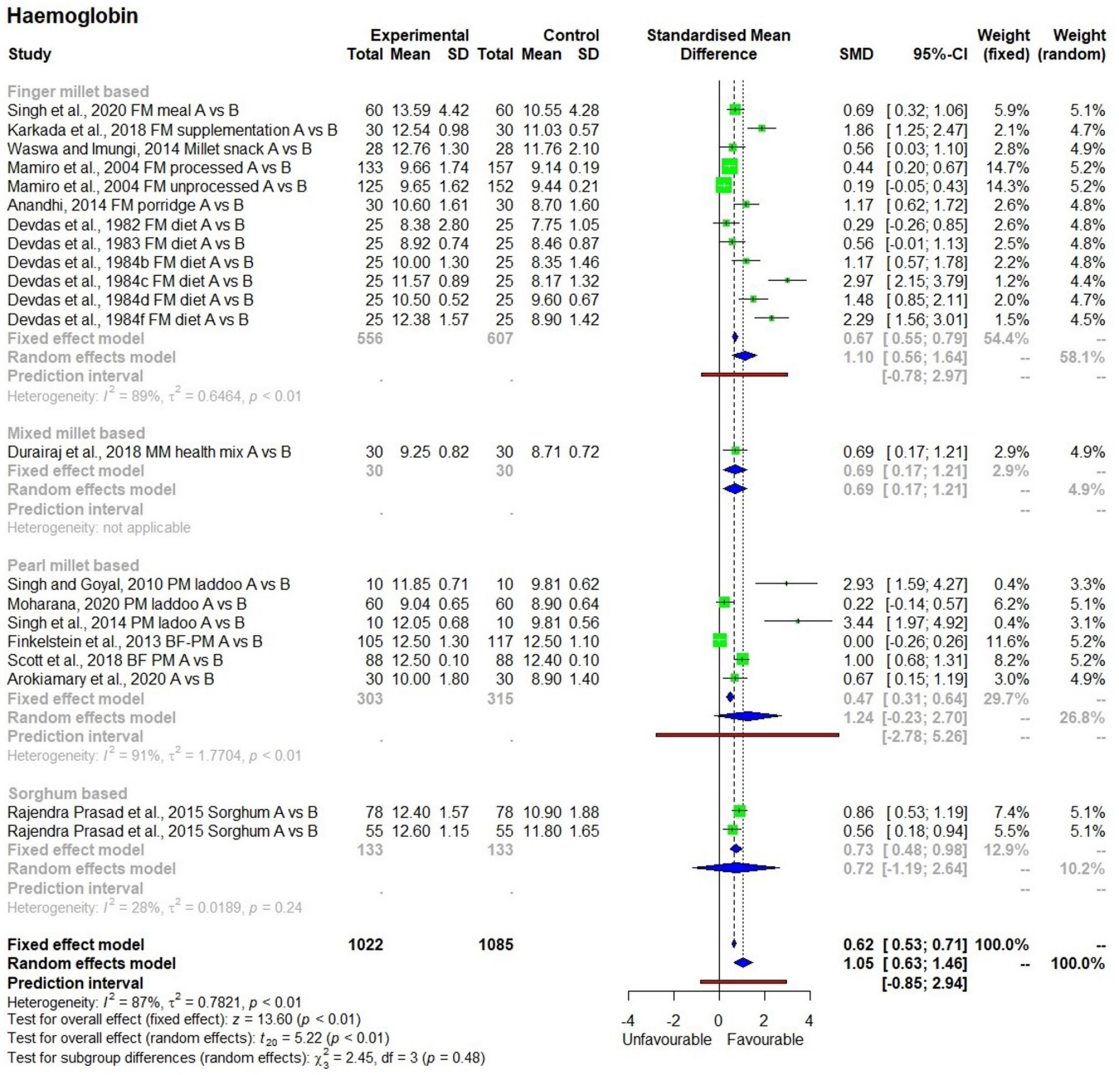
Effect of consuming different types of millets on Hemoglobin level.

A subgroup analysis was performed to determine the effects of consumption of millets on hemoglobin levels based on the type of millets used in the study (finger millet, pearl millet, mixed millets, and sorghum), the duration of the study (‘short' or ‘long'), and the age group of the participants (children, adolescents, and adult). It was not possible to conduct a subgroup analysis based on the iron content of the grains since only three of the 19 studies on the effectiveness of millets on hemoglobin outcome indicated the iron content in millets used. A subgroup analysis conducted to study the effects of consuming different types of millets on hemoglobin levels showed no significant (Figure 8) difference due to using any particular type of millets (*p* = 0.48). Finger millet and pearl millet had similar effects on hemoglobin levels (*p* < 0.05). The effects of using mixed millets could not be estimated due to the small number of studies.

### Risk of Bias Assessment

The risk of bias assessment shows major risks coming from blinding of the assessment. As millets have a unique colour, texture, and size it is not possible to conduct the study by blinding the sample. However, it is possible to blind the proportion added and the type of millets included, among other things. The sample size in studies was reasonable, ranging from 10 to 133 samples for assessing the impact of millet consumption on hemoglobin levels. Except for two studies, all the studies had more than 10 samples each. The Trim and Fit method were applied to account for the effects of small sample size on studies until the funnel plot became symmetrical (*p* < 0.0001).

## Discussions

This study used the term ‘high iron millet' to indicate any millet that provides more than 6 mg iron/100 g of grain. Note that the four human interventions investigated were based on biofortified pearl millet. Biofortification is a process that increases the concentration of targeted nutrients in crops through breeding technologies and can be a promising, sustainable, and cost-effective approach to combating micronutrient deficiencies (59). The other human studies and *in vitro* studies used varieties of millets that were commonly available.

All the *in vivo* human studies (lasting 28 days to 6 months), except two studies, used 84 to 300 g of pearl millet or finger millet in the form of a meal: *bhakri*/flatbread, porridge, or *upma* (thick porridge prepared by seasoning and adding spices, with or without vegetables). The meal was provided once or twice a day. It diversified the cereal-based main meal by incorporating millets and increased the intake of iron from the main meal.

Among the 19 studies in the meta-analysis to assess the impact on hemoglobin levels, only 3 studies indicated the levels of iron in millets used. The iron content in the millet grain has a huge impact on bioavailable iron, which in turn impacts the anemia status. It is noteworthy that the consumption of iron-rich pearl millet with 8.3 mg/100 g of iron levels contributed more than 50% of iron to the entire meal with 14.1 ± 9 mg of iron and improved the iron bioavailability with bioavailable iron of 1 ± 0.4 mg/day, compared with the consumption of a low iron pearl millet (< 3 mg/100 g) meal. Regardless of the percentage of bioavailability of iron, if the millet contains high iron, then the amount of bioavailable iron also increases. In general, the percentage bioavailability of iron from plant-based food varies from 1 to 10% (60) and ICMR. (15) also shows that it considered 8% bioavailability of iron from cereal-based diets. This shows that compared with many plant-based foods, millets have an equal or better bioavailability percentage.

However, the same was not the case in *vitro* studies, probably because of the variation in the methodology used.

### *In vitro* Methods Used for the Bioavailability Study

Unlike *in-vivo, in-vitro* studies showed significant variation in the bioavailability percentage, with high iron millets having a lower average bioavailability percentage than moderate and low iron millets. However, the bioavailability percentage was generally high, averaging 17.64 ± 12.49 in the *in vitro* studies.

Taking bioavailability percentage into account, bioavailable iron varied significantly by variety and can approximately triple the quantity of iron bioavailable, ranging from 0.54 ± 0.21 in low iron varieties to 1.58 ± 1.24 in high iron varieties.

The huge variation in bioavailability percentage may be explained by the heterogeneity of the four methods adopted in the *in vitro* studies, which were iron solubility, iron dialysability, HCl-extractability, and gastrointestinal models. The *in vitro* methods aimed at mimicking the gastric and small intestinal phase involving the use of pepsin and pancreatin in iron solubility, dialysability and gastrointestinal methods. However, the studies of (49, 57) used HCl-extractability, which did not involve the use of pepsin and pancreatin and may have led to underestimation or overestimation since it did not consider the digestion of minerals in two key areas of the gastrointestinal tract, which distinguishes this method from the three others. The three other *in-vitro* methods also differed in the setup and conditions, which may have contributed to the differential outcome. The *in vitro* study by (50) used two approaches in the iron solubility method in which the usage of either HCl-pepsin or pepsin-pancreatin on mineral extractability, showed differences in the percentage of bioavailability. The differences can be explained by the endpoint measurements. The HCl-pepsin method measured the iron bioavailability at the end of the gastric phase with pH between 1 and 3 and the pepsin-pancreatin method measured the iron bioavailability at the end of the small intestinal phase with pH between 7 and 8. The differences in pH in these two stages may affect the iron solubility and hence the percentage of bioavailability. However, the authors did not outline the methods clearly. Four studies by (47, 53, 55, 56) used the iron dialysability method which was based on equilibrium dialysis. In the absence of *in vivo* epithelial uptake, this *in vitro* method adopted dialysis as a physical separation technique whereby a dialysis membrane was used. It is important to note that the selected molecular cut-off of the dialysis membrane, final pH adjustment, time of incubation, and the method used for iron quantification (colourimetric assay or spectrophotometry) are instrumental for consistency in the results (61). In addition, enzymes' sources, pH and digestion time, are also important parameters for standardization and may alter enzyme activities and possibly the results. The studies by (48, 54, 58) used gastrointestinal models and adopted different approaches in terms of simulated digestion fluids which influence the ionic strength and ratio of samples to buffer. The study of (54), adopted a more recent standardized static *in vitro* digestion model proposed by (62) which is useful to compare results of the inter and intra laboratory. Nevertheless, data validation between *in vitro* and *in vivo* studies may provide information when similar meals and experimental conditions are compared (61). Therefore, *in vitro* methods are useful screening tools to assess iron bioavailability involving a large number of food samples.

Post-harvest iron fortification of pearl millet (artificial fortification) increases the amount of iron available and can be added in large quantities to increase by 32% the total quantity of iron absorbed compared with naturally high iron pearl millet (44). However, a feasible and sustainable approach for long-run implementation would be to release more high iron millet varieties compared with any other method such as fortification and tablet supplementation. The studies showed that, based on the age group, 75 to 100% of the physiological requirement can be achieved through a standard meal prepared using high iron millets. It is noted that fortified foods have processing difficulties such as higher costs of processing and the use of artificial additives for post-harvest fortification. Given that there are many naturally occurring high iron millets, it is important to use them in efficacy studies to generate more science-based evidence and to enable the formulation of policies that would make them available to farmers and also increase the choices for consumers.

Processing had a significant positive impact on the bioavailability of iron (Table 3). Of the household and traditional processing methods, fermentation was found to be superior to all other processes for increasing bioavailable iron. Of the commercial processing methods, expansion was found to be superior compared with all other processing methods for increasing bioavailable iron. Generally, dietary inhibitory factors affect the efficiency of iron absorption (63). The major dietary inhibitory factors for iron absorption in millets are phytates and tannin. It is worth noting that millets have similar or lower levels of these anti-nutrients compared with common staples and legumes (5), which are further reduced by processing to positively improve the bioavailability of iron. Studies also showed significantly increased iron bioavailability in millets following different methods of processing. In addition, ascorbate (Vitamin C) in the presence of tannins decreases the chelating properties of tannins and thus increases the bioavailability of iron by up to 6.8 times (50). While one study showed that germination reduced tannin content by 50%, another study revealed a sixfold increase in the bioavailability of iron after the same process. The study of (64) demonstrated in *in vitro* studies that processing eliminated inhibitory factors such as phytates and increased the bioaccessibility of iron. However, *in vivo* studies are required to ascertain if similar effects are achieved using processed millets.

Considering the nutritional quality of all types of millets and their versatile nature of fitting into popular recipes using rice (3), replicating these studies with millets will be useful to identify variation in iron bioavailability and its benefits in reducing IDA.

## Limitation

Iron contamination of food from post-harvest treatment, storage, and cooking vessels, which could increase the iron content of the grain, was not reported by any studies included in this systematic review. However, it was noted that there have been many studies that do specifically look at or incorporate the impacts of iron contamination from external sources on a variety of different foods (65–67). Especially while reporting high iron levels (>10 mg/100 g), it is important to look at the contamination from an external source (44) to avoid inflated data.

## Conclusion

The systematic review and meta-analysis showed millets are an excellent source of iron with low-cost potential for reducing iron deficiency anemia. This underlined the need for policymakers to recognize the right varieties and types of millets rich in iron for use as supplement food to counter the high prevalence of anemia in many countries. Selecting the iron rich millet varieties and developing iron-biofortified millet that can provide additional bioavailable iron could be a promising approach to combatting IDA. Incorporating millets as a staple across Asia and Africa could have the potential to make a significant impact on IDA. This can also be applicable in communities where millets are traditional foods but not consumed regularly and access to alternative foods is limited. It can also be concluded that the bioavailability of iron in millets can be further improved through processes such as soaking, germination, decortication, and fermentation which can serve as an effective strategy to reduce iron deficiency anemia.

## Data Availability Statement

The original contributions presented in the study are included in the article/Supplementary Material, further inquiries can be directed to the corresponding author.

## Author Contributions

SA and JK-P: conceptualization. JK-P: resource. SA, JK-P, RKB, SU, and MV: data collection, screening, extraction, and analysis. SA, JK-P, IDG, NLBS, and SU: drafting original manuscript. TL, AR, TWT, KS, DJP, and RKB: critical reviewing of protocol and manuscript and editing. All authors contributed to the article and approved the submitted version.

## Funding

This research was supported financially by the Smart Food endowment fund.

## Conflict of Interest

The authors declare that the research was conducted in the absence of any commercial or financial relationships that could be construed as a potential conflict of interest.

## Publisher's Note

All claims expressed in this article are solely those of the authors and do not necessarily represent those of their affiliated organizations, or those of the publisher, the editors and the reviewers. Any product that may be evaluated in this article, or claim that may be made by its manufacturer, is not guaranteed or endorsed by the publisher.
